# Tuberculosis Drug Resistance Mutation Database

**DOI:** 10.1371/journal.pmed.1000002

**Published:** 2009-02-10

**Authors:** Andreas Sandgren, Michael Strong, Preetika Muthukrishnan, Brian K Weiner, George M Church, Megan B Murray

## Abstract

Andreas Sandgren and colleagues describe a new comprehensive resource on drug resistance mutations in*M. tuberculosis*.

## Resistant TB Is a Serious Threat to Global Health

Tuberculosis (TB) remains the leading cause of death from a largely preventable and curable infectious disease, with an estimated 1.7 million deaths in 2006 [1]. Global prospects for TB control are challenged by the emergence of drug-resistant strains, especially those that are multidrug resistant (MDR) and extensively drug resistant (XDR) [2].

Soon after anti-TB drugs became available in the 1940s came reports of drug resistance among patients undergoing treatment [3]. With the advent of “short course chemotherapy” in the 1980s, the duration of treatment fell from 24 to six months, but even then full adherence to treatment regimens has been difficult to accomplish, due to the extensive length of therapy necessary to achieve cure. The prevalence of TB resistant to a single drug was continuously on the rise in several parts of the world, and eventually in the early 1990s, multiple converging factors led to an explosive emergence of MDR-TB, defined as resistance to the two most effective first-line anti-TB agents, isoniazid and rifampicin.

The most recent estimates on the prevalence of anti-TB drug resistance come from surveys conducted by the World Health Organization and the International Union Against Tuberculosis and Lung Disease. These organizations investigated both new and previously treated TB cases in 93 geographical settings between 2002–2006 [4]. In these surveys, the prevalence of MDR-TB ranged from 0% to 22% among newly diagnosed cases and from 0% to 60% among previously treated cases. In addition, since 2002, 45 countries have reported cases of XDR-TB, i.e., TB that is resistant not only to isoniazid and rifampicin but also to at least one fluoroquinolone and to any of the following injectable second-line drugs: kanamycin, amikacin, or capreomycin. Of the MDR isolates tested for second-line drugs, 0%–30 % were found to be XDR [4].

**Summary Points**

- The emergence and spread of drug-resistant TB threatens global TB control efforts.
- There is an urgent need for new diagnostic tests that rapidly identify drug sensitivity profiles of M. tuberculosis strains and for new drugs to treat resistant disease.
- We have established a comprehensive database listing mutations associated with TB drug resistance and the frequency of the most common mutations associated with resistance to specific drugs.
- We have made this interactive database publicly available on the Web site http://www.tbdreamdb.com/ so that it can serve as a resource for the development of molecular diagnostics for TB.
- Other uses of the database include structural mapping of mutations to study mechanisms of resistance for drug discovery purposes.

## Identification of Drug Resistance Mutations in TB

Drug resistance in TB is believed to be mediated exclusively by chromosomal mutations, which affect either the drug target itself or bacterial enzymes that activate prodrugs. Since the early 1990s, numerous studies have described the genetic mechanisms of drug resistance in Mycobacterium tuberculosis, and a wealth of data has accumulated on the mutations found in isolates resistant to specific drugs [5]. For some drugs, like isoniazid and rifampicin, a large number of mutations have been identified that confer resistance, and these mutations account for most of the resistance found among clinical isolates. For other drugs, such as streptomycin and many of the second-line drugs, known drug resistance mutations occur in only a small proportion of resistant isolates [5]. As resistance has become even more prevalent, and high-throughput sequencing methods and genotyping strategies have been developed and refined, large-scale studies to identify the mutations associated with resistance have been undertaken throughout the world. Until now, there has been no single resource where such drug resistance mutation data are readily available to use in research and development. In order to propel the use of the data that are scattered over a large number of publications, many of which are not readily accessible to researchers around the globe, we have compiled the data available on drug resistance mutations in M. tuberculosis in an open-access database that can be used for a diverse array of purposes.

## Applications of the Database

Centralized databases, curated from the literature or high-throughput experiments [6,7], have helped usher in the era of systems biology [8], and have helped researchers identify global trends and important biological features that would have only been apparent through a comprehensive analysis of the combined data [9]. Examples include protein–protein interaction databases [10,11], genomic databases [12,13], and microarray databases [14–16]. Through this project we present a new database devoted to drug resistance mutations in TB, called the TB Drug Resistance Mutation Database (TBDReaMDB). By providing a comprehensive, single resource of drug resistance mutations in TB, we hope to accelerate and encourage new discoveries that will have applications ranging from diagnostics to drug discovery. We envision that this database will expand as additional mutations are identified in the coming years and will serve as a platform for diverse analyses and projects. TBDReaMDB also complements other TB-related databases such as TBDB (http://www.tbdb.org/), a recently developed Web-based resource housing both annotated genome sequence data and expression data from M. tuberculosis and related species [17].

### Resource for sequencing projects.

The creation of this database was originally precipitated by our in-house need for information on known mutations found in drug-resistant TB, to be used in a large-scale whole-genome sequencing project of MDR- and XDR-TB strains [18]. Whole-genome sequencing identifies a large number of mutations among isolates from diverse geographic regions that exhibit varying degrees of drug resistance. The data in the mutation database can be used to screen the mutations discovered by sequencing, thereby allowing the user to distinguish mutations and genes that previously have been associated with resistance from newly identified resistance-associated mutations, or random mutations that serve as markers of the genetic lineage of the particular strain.

### Development of diagnostics.

New tools for rapid and accurate diagnosis of drug-resistant TB are urgently needed. Sequence-based diagnostic methods have been developed that detect specific mutations associated with drug resistance. These tools have the advantage of being rapid, high-throughput, and easily compared between laboratories. However, the development of such diagnostic tools relies on detailed information about the mutations that lead to drug resistance and their relative frequency. Currently the two main diagnostic tests available commercially are the INNO-LiPA TB test (Innogenetics) [19] and the GenoType MTBDR*plus* kit (Hain Lifescience) [20]. These assays have recently been approved by the World Health Organization as a tool for rapid MDR-TB diagnosis [21]. These “line probe assays” are based on the detection of a set number of mutations in a few genes associated with resistance against isoniazid and rifampicin. These assays may therefore be unable to detect all isoniazid and rifampicin resistance and do not detect resistance to other first-line or second-line drugs. This database project establishes a foundation that we hope will spur future development of assays with improved sensitivity and specificity and the expansion of these tools to detect resistance to other first- and second-line drugs. In addition, the data may also be used in future research to understand the clinical importance of rare mutations.

### Geographical distribution and surveillance of mutations.

Differences in the type and frequency of mutations in drug-resistant isolates in diverse geographical regions can pose challenges for the development of a sequence-based diagnostic, especially if the test relies on detecting a limited number of mutations. Different drugs of the same drug class may also interact differently with the molecular drug targets and thus affect the mutations selected for [22,23]. In addition, some specific mutations have been shown to lead to high-level resistance, while others confer low-level resistance to a specific drug [24]. Others may lead to cross-resistance to drug analogs [25]. Regional differences in drug analogs used have been shown to select for different drug resistance mutations; for example, mutations found in regions that use specific rifamycin analogs differ from those in which other analogs are used [26]. The mutations that arise from these different drug analog exposures may help explain some of the reported geographical differences in drug efficacy [26]. We have therefore included the geographic sites of resistance studies in our database. As more data on the frequency of mutations become available through large-scale sequencing projects, we will garner an even more comprehensive understanding of the global distribution of specific mutations and a better perception of the role of geographical differences.

### Drug discovery.

The database can also be used for novel computational technologies in drug discovery. Mutations may be mapped onto protein structures in order to better understand the mechanisms of resistance at the molecular and atomic level. Such analyses may help us to better understand how particular mutations lead to resistance, how mutations impact the affinity or activation of corresponding drugs, and how mutations may impact the fitness of the bacteria. Trends, such as correlated or synergistic mutations, may also become apparent through the comprehensive analysis of the data on mutations associated with resistance. In addition to revealing the mechanisms of resistance on a global scale, the resulting mapping of mutations may also suggest drug modifications or alternative strategies to counter emerging drug resistance in TB.

## A New Comprehensive Database

TBDReaMDB is a comprehensive resource on drug resistance mutations in M. tuberculosis. We conducted a systematic review (see Text S1 for a brief description of the systematic review strategy) to identify drug resistance mutations from the existing literature to include in the database. We chose not to make any a priori decisions as to whether the mutations described in the literature actually confer drug resistance or are possible secondary compensatory mutations, but instead included all mutations that have been described in drug-resistant strains more often than drug-sensitive isolates. For each mutation, the database provides complete codon changes for each mutation at both the nucleotide and amino acid level.

### Systematic compilation of mutations found in clinical isolates of drug-resistant TB.

The database is divided into two parts. The first part lists all the unique mutations reported in drug-resistant TB isolates, as well as information on the time period of isolate collection, country of origin, molecular detection method, resistance pattern, and susceptibility testing method. Since many of the mutations are reported in multiple publications, we included only the first report that identified a specific mutation. As of September 1, 2008, TBDReaMDB contains 946 unique mutations associated with seven different drug classes and spread over 36 genes, two intergenic/promoter regions, and one ribosomal RNA coding region.

### Frequency of high-confidence mutations associated with drug resistance in TB.

The second part of the database provides data on the relative frequency of the most common mutations associated with resistance to specific drugs, as reported in surveys from diverse geographical sites. For each drug, we extracted data from ten high-quality publications that reported the frequency of mutations associated with resistance to that drug. In addition to the information about the frequency of specific mutations among resistant and sensitive isolates, we have also extracted the type of article-specific data, including time period of isolate collection and country of origin. Such frequency data might be useful for the design and validation of sequence-based diagnostic tools to estimate the number of genes or mutations needed to develop a sensitive and specific assay. These data may also serve as a baseline for surveillance of geographical and temporal trends in the prevalence of specific mutations, as well as for research on the implications of geographical differences in mutation frequencies for development of new diagnostic tools.

## Open Access through an Interactive Web Site

We have created an interactive Web site that allows users to visualize all the specific mutations associated with resistance to each drug (http://www.tbdreamdb.com/). For more information on the TBDReaMDB Web site, please see Box 1. We envision that this Web site, in addition to presenting the literature in a manually curated database, also will serve as a gateway to post data from future research and development undertaken by its users. In keeping with the scientific philosophy of open access to the public, the database is open to the public and free under the Creative Commons Attribution 3.0 Unported License (http://creativecommons.org/licenses/by/3.0/). We hope that the Web site will be a portal to communicate future research on drug resistance mechanisms, and to spur development of novel rapid diagnostic tools and drug discovery.

**Box 1. The TBDReaMDB Web Site**

**Figure 1:**
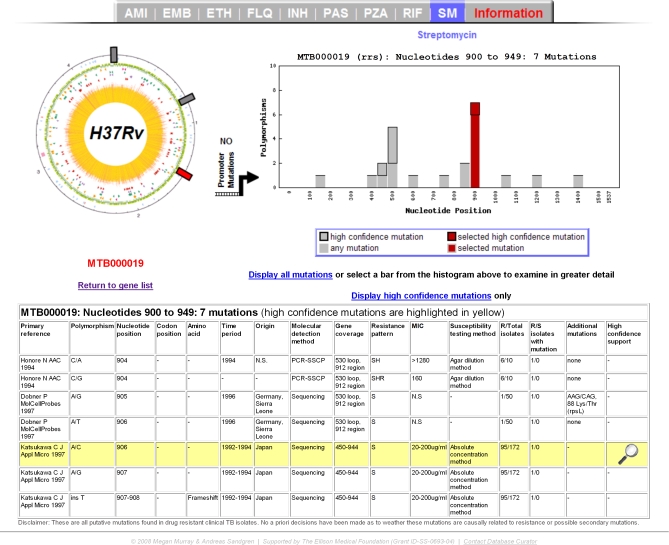
Part of the Interface of the Web Site This view shows the distribution of mutations in rrs (MTB000019) associated with streptomycin resistance.

The TBDReaMDB Web site (http://www.tbdreamdb.com/) was launched on September 1, 2008. All the data collected as part of this study are presented on the Web site and accessible to the public. The data are free of usage but recognition of the data source is required; this article should be referred to as well. The Web site is organized by drug and presents all the information about the mutations described in the literature. The mutations presented have been color coded to highlight the high-confidence mutations for which frequency data are available. For an example of the interface of the Web site, see Figure 1. The data are also accessible in the form of global Excel and tab-delimited spreadsheets for all mutations. The database developed through this project has been constructed to allow continuous updates as additional mutations in clinical TB isolates are reported. To submit new mutations or to give us feedback you may e-mail us at the address found on the homepage of the Web site. On the Web site you can also find all the criteria that must be fulfilled for the mutations to be qualified for inclusion in the database.

## Future Directions

The current version of TBDReaMDB, as of September 1, 2008, is based on literature available up to January 2008. The database will be kept updated and manually curated to provide an accurate picture of the current distribution of mutations associated with drug resistance in TB. To maintain the database up to date, we will include new information submitted to the mutations lists and mutation prevalence data sets.

### Submission of new mutations or frequency data.

We will include novel mutations found in clinical isolates of drug-resistant TB if they (1) are from published studies of clinical M. tuberculosis isolates, (2) occur in isolates that have been characterized by phenotypic drug sensitivity testing, and (3) are identified by specification of the gene, nucleotide position, and the nucleotide and/or amino acid change. Mutations that meet these criteria may be submitted through the Web site and will be added continuously after review.

For studies to be included in the database describing the frequency of common mutations associated with drug resistance in M. tuberculosis, they must (1) be studies of clinical M. tuberculosis isolates; (2) have large sample sizes (a minimum of 100 resistant isolates are required for isoniazid and rifampicin; for the other drugs sample sizes have been determined empirically depending on the ten largest studies published so far—see Web site); (3) report on phenotypic drug sensitivity testing for all isolates; (4) use validated methods to identify drug resistance mutations; (5) identify the nucleotide position and the nucleotide change; and (6) specify the number of resistant and sensitive isolates carrying a specific mutation. The frequency database will be updated regularly with the most recent data.

### Posting of results from future applications for open access.

We will customize the Web site to house results and references from projects using the information available through our resource. Scientists who wish to post data on the Web site may submit an inquiry for space to present results from data analysis, graphics, or links to their own Web site or published articles.

## Conclusions

This project is meant to equip the efforts on research and development of novel technologies and approaches needed for the global surveillance and control of drug-resistant M. tuberculosis, as well as for the diagnosis and clinical care of individual patients with this disease. We have assembled a comprehensive database of putative and well-established resistance mutations and presented the relative frequency and geographical distribution of the most common high-confidence mutations. TBDReaMDB is currently the largest and only open-access database for mutations associated with drug-resistant TB. The most important aspect of this project is that all the data are readily available for open access through a publicly accessible Web site. These data constitute an important resource for the TB research community and others to spur further research on drug resistance mechanisms that eventually may lead to the development of novel diagnostic tools and therapeutics to combat resistant TB.

## Supporting Information

Text S1 Systematic Literature Review: Methods (48 KB DOC).

## Abbreviations

MDR: multidrug resistant
TB: tuberculosis
TBDReaMDB: TB Drug Resistance Mutation Database
XDR: extensively drug resistant
