# Supplementary material for: Tuberculosis Drug Resistance Mutation Database
Source: PLoS Med. 2009 Feb 10;6(2):e1000002. doi: 10.1371/journal.pmed.1000002 (PMC2637921; doi:10.1371/journal.pmed.1000002)
Supplement: Supplementary file 1 [file pmed.1000002.sd001.doc]

**Systematic literature review – Methods**

This gives a brief description of the search strategy, inclusion and exclusion criteria, and data extraction used to obtain the data of the database.

*Data sources*

We searched the MEDLINE and EMBASE databases (Jan 1966 – Feb 2008) for studies that reported an association between specific mutations in clinical isolated of *M. tuberculosis* and phenotypic resistance to Isoniazid, Rifampin, Streptomycin, Pyrazinamide, Fluoroquinolones, Ethambutol, Kanamycin, Amikacin, Capreomycin, Ethionamide, and para-aminosalisylic acid (PAS). We also searched bibliographies of identified reports and reviews for additional references. We considered articles published in any language.

*Search terms*

For the comprehensive list of mutations, combinations of the following search terms were used:

Tuberculosis AND (mutation OR mutations) AND (isoniazid OR rifampin OR streptomycin OR aminoglykosides OR pyrazinamide OR fluoroquinolones OR ethambutol OR kanamycin OR amikacin OR capreomycin OR ethionamide OR PAS OR katg OR ahpc OR inha OR oxyr OR rpob OR rrs OR rpsl OR gidb OR emba OR embb OR embc OR pnca OR gyra OR gyrb)

For the frequency distribution of common mutations associated with drug resistance in TB, combinations of the following search terms were used:

INH: tuberculosis AND (mutation OR mutations) AND (isoniazid OR katg OR ahpc OR inha OR oxyr); RIF: tuberculosis AND (mutation OR mutations) AND (rifampin OR rifampicin OR rpob); SM: tuberculosis AND (mutation OR mutations) AND (streptomycin OR rrs OR rpsL OR gidb); EMB: tuberculosis AND (mutation OR mutations) AND (ethambutol OR emba OR embb OR embc); PZA: tuberculosis AND (mutation OR mutations) AND (pyrazinamide OR pnca); FLQ: tuberculosis AND (mutation OR mutations) AND (fluoroquinolones OR oxifloxacin OR moxifloxacin OR levofloxacin OR ciprofloxacin OR gyra OR gyrb); AG: tuberculosis AND (mutation OR mutations) AND (aminoglykosides OR kanamycin OR amikacin OR capreomycin OR rrs OR tlya)

*Study selection*

A study was included in the database if: 1) it was a study of clinical *M. tuberculosis* isolates; 2) drug sensitivity testing had been performed on all isolates tested for mutations; 3) the nucleotide position or codon position and the nucleotide change or amino acid change was given. For the part of the database containing frequency distribution data we also required that: 4) the number of resistant and sensitive isolates were given for each specific mutation; 5) that drug sensitive isolates had been tested as controls; 6) the sample size was sufficient (the minimum sample size was set to 100 resistant isolates for INH and RIF. For the other drugs the minimum sample size depended on the number of resistant isolates for a particular drug. We selected the ten most recent articles to maximize the number of resistant isolates included. If more than one article had the same number of resistant isolates we selected the last published article).

A study was excluded from the frequency distribution database if: 1) it was a review, letter or editorial work; 2) the article had been withdrawn or citation information incorrect; 3) the information about mutations or number of isolates was not sufficient to extract the data described below; 4) isolates were selected based on prior knowledge about mutations; 5) novel detection methods were used without confirmation by direct sequencing; 6) the study tested for only a single mutation; 7) drug sensitivity testing was not performed; 8) the collection of isolates had already been analyzed in another study selected for inclusion.

*Data Extraction*

We extracted and recorded information on: the gene in which putative resistance mutations were found; the nucleotide position and/or codon position; nucleotide change and/or amino acid change; drug sensitivity pattern; drug sensitivity testing method used; molecular detection method of mutations; part of the gene covered in the mutation testing; geographic origin of collected isolates; and, time period of isolate collection. For each study eligible to be included in the frequency distribution database, we collected information on mutations that occurred either in at least 5% of the drug resistant isolates in any of the studies included, or that were reported in resistant isolates in more than one of the studies included. Additional information collected for the frequency database included total numbers of resistant and sensitive isolates; the number of drug resistant and drug sensitive isolates carrying a specific mutation.
